# Supplementary material for: Are the Consumption Patterns of Sports Supplements Similar among Spanish Mountain Runners?
Source: Nutrients. 2023 Jan 4;15(2):262. doi: 10.3390/nu15020262 (PMC9866651; doi:10.3390/nu15020262)
Supplement: Supplementary file 1 [file nutrients-15-00262-s001.zip › nutrients-2088709-supplementary_revised.pdf]

# ESTUDIO DEL CONSUMO DE SUPLEMENTOS NUTRICIONALES EN CORREDORES POR MONTAÑA

Las respuestas a este cuestionario servirán para valorar y cuantificar el uso de suplementos en su deporte. Los resultados colectivos del cuestionario nos ayudarán a describir el patrón de consumo de suplementos en CxM y plantear recomendaciones sobre el consumo de los mismos.

Se trata de un cuestionario CONFIDENCIAL. Toda la información será analizada por personal técnico sujeto al secreto profesional y utilizada exclusivamente para los fines descritos. La Ley Orgánica 3/2018 de Protección de Datos será respetada en todo momento. Este estudio ha sido aprobado por el comité de ética de la Universidad de Alicante (#UA 2021-02-01). En el informe de resultados no podrán ser identificadas las respuestas de ninguna persona de forma individualizada.

Para participar en el estudio es necesario ser mayor de edad y contestar INDIVIDUALMENTE. Por ello te pedimos que respondas siguiendo las instrucciones de cada pregunta para contestar. Es muy importante que contestes TODAS LAS PREGUNTAS ya que muchas se tratan conjuntamente, de manera que si dejas alguna pregunta sin contestar es probable que otras que hayas contestado se anulen y se pierda la información para el análisis de resultados.

Para llevar a cabo esta investigación se ha creado un Grupo de Trabajo formado: Noelia Rubio (noeliarubio.dn@gmail.com), Rubén Jiménez (rja10@gcloud.ua.es), Isabel Sospedra (isospedra@ua.es) y José Miguel Martínez (josemiguel.ms@ua.es). Si necesitas realizar alguna consulta te puedes dirigir a cualquiera de sus miembros.

MUCHAS GRACIAS POR TU COLABORACIÓN

CONSENTIMIENTO:
- Soy mayor de edad.
- He sido informado/a de las características del Proyecto de Investigación titulado: "Estudio del consumo de suplementos nutricionales en CxM".
- He leído la información del proyecto, y he podido formular las dudas que me han surgido al respecto.
- Considero que he entendido dicha información.
- Estoy informado/a de la posibilidad de retirarme en cualquier momento del estudio.
- Estoy informado del modo en que serán tratados mis datos.
- No rellenar más de una inscripción por persona y escribir en MAYÚSCULAS todas las respuestas que requieran texto.

*Obligatorio

1. En virtud de tales condiciones ¿consientes participar en este estudio? *

   *Marca solo un óvalo.*

   - Si
   - No

DATOS INICIALES

2. SEXO *

   *Marca solo un óvalo.*

   - Hombre
   - Mujer
   - No sabe / no contesta

3. Fecha de nacimiento *

   *Ejemplo: 7 de enero del 2019*

4. Edad actual en años *

   Indicar edad en números

5. Comunidad Autónoma de residencia *

*Marca solo un óvalo.*

- Andalucía
- Cataluña
- Comunidad de Madrid
- Comunidad Valenciana
- Galicia
- Castilla y León
- País Vasco
- Canarias
- Castilla-La Mancha
- Región de Murcia
- Aragón
- Isla Baleares
- Extremadura
- Principado de Asturias
- Navarra
- Cantabria
- La Rioja
- Melilla
- Ceuta

6. ALTURA APROXIMADA EN CENTÍMETROS (cm) *

En centímetros

7. PESO APROXIMADO EN KILOGRAMOS (kg) *

En kilogramos

8. % APROXIMADO DE GRASA (Solo si se sabe la respuesta)

Porcentaje

PRÁCTICA DEL DEPORTE

9. ¿Estás federado actualmente? *

*Marca solo un óvalo.*

- Sí
- No

10. INDICA EL NÚMERO DE AÑOS APROXIMADO QUE LLEVAS FEDERADO *

*Marca solo un óvalo.*

- No estoy federado
- 1 año
- 2 años
- 3 años
- 4 años
- 5 años
- 6 años
- 7 años
- 8 años
- 9 años
- 10 o más años

11. INDICA EN EL NIVEL QUE COMPITES ACTUALMENTE *

Señala el de mayor categoría

*Marca solo un óvalo.*

- Provincial o autonómico
- Nacional
- Europeo
- Mundial
- Olímpico
- Otro: ____________________

12. INDICA LOS AÑOS QUE LLEVAS COMPITIENDO EN EL NIVEL SEÑALADO ANTERIORMENTE *

*Marca solo un óvalo.*

- 1 año
- 2 años
- 3 años
- 4 años
- 5 años
- 6 años
- 7 años
- 8 años
- 9 años
- 10 o más años

## ENTRENAMIENTO Y COMPETICIÓN

13. ¿CUÁNTOS DÍAS DE MEDIA DEDICAS AL ENTRENAMIENTO A LA SEMANA? *

APROXIMADAMENTE

*Marca solo un óvalo.*

- 1 día
- 2 días
- 3 días
- 4 días
- 5 días
- 6 días
- 7 días

14. ¿CUÁNTO TIEMPO, APROXIMADAMENTE, DEDICAS CADA DÍA AL ENTRENAMIENTO? *

Elige el intervalo de tiempo que más se ajuste a tu tiempo de entrenamiento

*Marca solo un óvalo.*

- <30 minutos
- Entre 30 minutos y 1 hora
- Entre 1 hora y 1,5 horas
- Entre 1,5 horas y 2 horas
- >2 horas

15. SUELES ENTRENAR... *

Elige el intervalo de tiempo que más se ajuste a tu tiempo de entrenamiento

*Marca solo un óvalo.*

- Por la mañana
- Por la tarde
- Por la mañana y la tarde
- Otro: ____________________

16. ¿CUÁNTAS COMPETICIONES TIENES AL AÑO? *

Elige la opción que más se acerque a la realidad

*Marca solo un óvalo.*

- 1
- 2
- 3
- 4
- 5
- 6
- 7
- 8
- 9
- 10
- 11
- 12
- más de 12
- Sin frecuencia exacta
- No sabe no contesta

17. ¿EN QUÉ DISTANCIA DE CARRERAS SUELES COMPETIR? (Indicar en la que más participes. Si es Otra distancia, indicar distancia)

*Selecciona todos los que correspondan.*

- <21km
- Media Maratón (21 km)
- Entre 21 y 34 km
- De 34 a 42km
- Maratón de Montaña (42km)
- Ultramaratón (>42km en un solo día)
- Ultradistancia (>80km en un solo día)
- Ultramaratón por etapas (>42km durante varios días seguidos)
- Otro: ____________

18. SUELES COMPETIR... *

Elige el intervalo de tiempo que más se ajuste a tu tiempo de entrenamiento

*Marca solo un óvalo.*

- Por la mañana
- Por la tarde
- Por la mañana y la tarde
- Otro: ______________________

DIETA

19. ¿SIGUES EN LA ACTUALIDAD ALGÚN TIPO DE DIETA O FORMA CONCRETA DE ALIMENTARSE? *

*Marca solo un óvalo.*

- Si *Salta a la pregunta 20*
- No *Salta a la pregunta 23*

DIETA CONTINUACIÓN

20. ¿QUÉ TIPO DE DIETA ES? *

*Marca solo un óvalo.*

- Vegetariana
- Vegana
- Mediterránea
- Ovolactovegetariana
- Paleo
- Disociada
- Macrobiotica
- Flexible
- Cetogénica
- Otro: ____________________

21. ¿POR QUÉ LA HACES? *

*Marca solo un óvalo.*

- Para cuidar tu salud
- Por estética
- Por enfermedad
- Por rendimiento
- Por religión o creencias
- Otro: ____________________

22. ¿TE LLEVA ALGUIEN O TE APOYAS EN ALGO PARA LLEVAR LA DIETA? *
EN CASO AFIRMATIVO INDICA LA RESPUESTA ADECUADA.

*Marca solo un óvalo.*

- No
- Dietista-Nutricionista
- Médico
- Farmaceutico
- Entrenado Personal
- Fisioterapeuta
- Alguien sin ningula titulación
- Me apoyo en un libro
- Me apoyo en un blog
- Me apoyo en un amigo
- Me apoyo en Redes Sociales
- Otro: ____________________

## SUPLEMENTOS

23. ¿ESTÁS A FAVOR DEL CONSUMO DE SUPLEMENTOS EN LA ACTIVIDAD FÍSICA DENTRO DE LA LEGALIDAD? *

*Marca solo un óvalo.*

- Si
- No
- No sabes/No contestas

24. ¿HAS CONSUMIDO EN ALGUNA OCASIÓN ALGÚN SUPLEMENTO NUTRICIONAL? *

*Marca solo un óvalo.*

- Si *Salta a la pregunta 25*
- No

SUPLEMENTOS CONTINUACIÓN

25. SEÑALA CUALES DE ESTOS SUPLEMENTOS CONSUMES DE MANERA HABITUAL *

ESTÁN ORDENADOS ALFABÉTICAMENTE. PUEDES SEÑALAR TANTOS CUANTOS CONSUMAS DE MANERA HABITUAL

*Selecciona todos los que correspondan.*

- [ ] 5-HTP (5-Hidroxitriptofano)
- [ ] ATP
- [ ] Ácido Aspártico
- [ ] Ácido Alfa Lipoico (ALA)
- [ ] Ácido Fosfatídico
- [ ] Ácido Hialurónico
- [ ] Ácidos Grasos ω-3
- [ ] Ácidos Grasos ω-6
- [ ] Ácidos Grasos ω-9
- [ ] Ácido Linoleico Conjugado (CLA)
- [ ] Aceite de Coco
- [ ] Aceite de Hígado de Bacalao
- [ ] Aceite de Lino
- [ ] Aceite de Onagra
- [ ] Aceite de Prímula
- [ ] Amilopectina
- [ ] Aminoácidos esenciales (EEAA)
- [ ] Aminoácidos ramificados (BCAA)
- [ ] Arginina
- [ ] Barritas Energéticas o Deportivas
- [ ] Barritas Proteicas
- [ ] Bebida Isotónica o de Reposición
- [ ] Beta Alanina
- [ ] Bicarbonatos
- [ ] Bloqueadores de Carbohidratos
- [ ] Cafeína (capsula, pastilla, liquido o chicle)
- [ ] Carbohidratos ("Gainers")
- [ ] Carnitina (Acetil-L-Carnitina)
- [ ] Carnitina (L-Carnitina)
- [ ] Cartílago de Tiburón
- [ ] Caseína Micelar
- [ ] Chitosan
- [ ] Ciclodextrinas
- [ ] Cinc
- [ ] Citrulina (Malato o L-citrulina)
- [ ] Complejo Mineral

- [ ] Complejo Vitamínico
- [ ] Colágeno
- [ ] Condroitina
- [ ] Creatina (Monohidrato)
- [ ] Creatina (Kre-Alkalyn)
- [ ] Creatina (Etil Éster)
- [ ] Curcumina
- [ ] Dextrosa
- [ ] Dimetilglicina
- [ ] Diuréticos
- [ ] Electrolitos (en polvo o pastillas)
- [ ] Epicatequina
- [ ] Epigalocatequina-3-galato (EGCG)
- [ ] Espirulina
- [ ] Fórmulas pre-entreno
- [ ] Fosfato de sodio
- [ ] Geles deportivos
- [ ] Ginseng
- [ ] Glicerol
- [ ] Glucosamina
- [ ] Glutamina
- [ ] Gominolas
- [ ] Greens
- [ ] Guarana
- [ ] Hidrolizado de caseína
- [ ] Hidroximetilbutirato (HMB)
- [ ] Hierro
- [ ] Isomaltulosa
- [ ] Jalea Real
- [ ] Lecitina de Soja
- [ ] Leucina
- [ ] Levadura de Cerveza
- [ ] Magnesio
- [ ] Maltodextrina
- [ ] Melatonina
- [ ] Mentol
- [ ] Metil-sulfonil-metano (MSM)
- [ ] Nitrato (zumo de remolacha)
- [ ] Nootrópicos
- [ ] N-Acetilcisteína
- [ ] Picolinato de Cromo
- [ ] Polen

- [ ] Potenciador de Testosterona
- [ ] Precursor Hormonal
- [ ] Probióticos
- [ ] Proteína de Carne
- [ ] Proteína de Suero (Whey Protein)
- [ ] Proteína Vegetal (soja, cáñamo…)
- [ ] Quinina
- [ ] Quercetina
- [ ] Recuperadores (mezcla de carbohidratos y proteínas)
- [ ] Ribosa
- [ ] Sinefrina (p-sinefrina)
- [ ] Suplemento de cereza ácida
- [ ] Suplemento de cetonas
- [ ] Taurina
- [ ] Teanina
- [ ] Teacrina
- [ ] Té verde (completo o extractos)
- [ ] Tribulus
- [ ] Tirosina
- [ ] Triglicéridos de cadena media (MCT)
- [ ] Vitamina C
- [ ] Vitamina D
- [ ] Vitamina E
- [ ] Vitamina K
- [ ] Yohimbina
- [ ] Zinc
- [ ] ZMA
- [ ] Otro: ____________________

Sin título

26. INDICA QUÉ DÍAS DE PRACTICA DEPORTIVA SUELES CONSUMIRLOS *

*Marca solo un óvalo.*

- Entrenamiento *Salta a la pregunta 25*
- Competición
- Entrenamiento y competición
- Periodo vacacional o de descanso
- En todos lo casos anteriormente mencionados
- Otro: ____________________

27. INDICA CUANDO SUELES CONSUMIRLOS *

*Marca solo un óvalo.*

- Antes de la práctica deportiva
- Durante la práctica deportiva
- Después de la práctica deportiva
- En todos los casos anteriores
- Indiferentemente
- Otro: ____________________

28. ¿CON QUÉ FIN LOS CONSUMES? *

*Selecciona todos los que correspondan.*

- [ ] Para cuidar tu salud
- [ ] Por problemas de salud
- [ ] Por necesidad
- [ ] Por obligación
- [ ] Para mejorar su aspecto físico
- [ ] Para buscar rendimiento deportivo
- [ ] Por paliar algún déficit de la dieta
- [ ] Otro: ____________________

29. ¿DONDE SUELES COMPRAR LOS SUPLEMENTOS? *

*Selecciona todos los que correspondan.*

- [ ] En una Farmacia
- [ ] En un Gimnasio
- [ ] A un Amigo
- [ ] A un Monitor
- [ ] A un Dietista-Nutricionista
- [ ] En un Herbolario
- [ ] En un Centro Comercial
- [ ] En una Parafarmacia
- [ ] En Internet
- [ ] En una Tienda especializada
- [ ] Otro: ____________________

30. ¿QUÉ/QUIEN TE MOTIVÓ A TOMARLOS? *

*Selecciona todos los que correspondan.*

- [ ] Compañero de equipo
- [ ] Entrenador
- [ ] Internet
- [ ] Monitor
- [ ] Publicidad
- [ ] Dietista-Nutricionista
- [ ] Revista especializada
- [ ] Televisión
- [ ] Preparador Físico
- [ ] Médico
- [ ] Amigo
- [ ] Influencer en redes sociales
- [ ] Otro: ____________________

31. EN GENERAL, ¿CREES QUE TE DIERON RESULTADOS? *

*Marca solo un óvalo.*

| | 1 | 2 | 3 | 4 | 5 | |
|---|---|---|---|---|---|---|
| NIN( | ◯ | ◯ | ◯ | ◯ | ◯ | MUCHO RESULTADO |

32. SI FUERA EL CASO, ESCRIBE ALGÚN/OS DE LOS SUPLEMENTOS QUE CREES QUE NO TE DIERON NINGÚN RESULTADO

33. SI FUERA EL CASO, ESCRIBE ALGÚN/OS DE LOS SUPLEMENTOS QUE CREES QUE SI TE DIERON RESULTADO

34. ¿HAS CONSUMIDO O CONSUMIRÍAS ALGÚN SUPLEMENTO QUE FUERA PERJUDICIAL PARA LA SALUD PERO QUE TE AYUDARA A CONSEGUIR TUS OBJETIVOS? *

*Marca solo un óvalo.*

- ◯ Si
- ◯ No
- ◯ No sabe/No contesta

35. EN EL CASO QUE LA PREGUNTA ANTERIOR SEA AFIRMATIVA, SEÑALA CUÁL/ES DE LA SIGUIENTE LISTA HAS CONSUMIDO O CONSUMIRÍAS

*Selecciona todos los que correspondan.*

- [ ] Esteroides anabolizantes
- [ ] Hormona de crecimiento
- [ ] Insulina
- [ ] Efedrina
- [ ] Pseudoanfetaminas
- [ ] Otro: ____________

36. INDICA, DESDE TU PUNTO DE VISTA, EL USO GENÉRICO DE ESTE TIPO DE SUSTANCIAS EN CARRERAS DE MONTAÑA *

*Marca solo un óvalo.*

| | 1 | 2 | 3 | 4 | 5 | |
|---|---|---|---|---|---|---|
| NIN( | ◯ | ◯ | ◯ | ◯ | ◯ | MUCHO USO |

Este contenido no ha sido creado ni aprobado por Google.

Google Formularios
